# Crizotinib-induced osteitis mimicking bone metastasis in a stage IV *ALK*-rearranged NSCLC patient: a case report

**DOI:** 10.1186/s12885-019-6486-3

**Published:** 2020-01-06

**Authors:** F. Guisier, N. Piton, M. Bellefleur, N. Delberghe, G. Avenel, E. Angot, O. Vittecoq, M. Ould-Slimane, H. Morisse-Pradier, M. Salaun, L. Thiberville

**Affiliations:** 1 Service de pneumologie, oncologie thoracique et soins intensifs respiratoires, CHU Charles Nicolle, Rouen, France; 2 LITIS QuantIF EA4108, Normadie Univ, Rouen, France; 3 INSERM CIC 1404, CHU Charles Nicolle, Rouen, France; 4 Service d’anatomie et cytologie pathologiques, CHU Charles Nicolle, Rouen, France; 5 Service de rhumatologie, CHU Charles Nicolle, Rouen, France; 6 Service d’orthopédie et traumatologie, CHU Charles Nicolle, Rouen, France

**Keywords:** *ALK*-rearranged NSCLC, ALK inhibitors, Crizotinib, Ceritinib, Drug toxicity, Osteitis

## Abstract

**Background:**

Targeted therapies are a standard of care for first-line treatment of Anaplastic lymphoma kinase (*ALK*)-rearranged non small cell lung cancer (NSCLC). Giving the rapid pace of drug discovery and development in this area, reporting of adverse effects of ALK inhibitors is crucial. Here, we report a case of osteitis induced by an ALK inhibitor mimicking bone metastasis, a previously undescribed side effect of crizotinib.

**Case presentation:**

A 31-year-old woman with stage IV *ALK*-rearranged NSCLC presented with back pain after 3 months of crizotinib treatment. Diagnostic work-up showed osteitis on the 4th and 5th thoracic vertebrae, anterior soft tissue infiltration and epiduritis, without any sign of infection. Spinal cord decompression, histological removal and osteosynthesis were performed. Histologic examination showed necrosis with abundant peripheral neutrophils, no microorganism nor malignant cell. Symptoms and Computarized Tomography-abnormalities rapidly diseappeared after crizotinib withdrawal and did not recur after ceritinib onset.

**Conclusions:**

This is the first report of crizotinib-induced osteitis. Crizotinib differs from other ALK inhibitors as it targets other kinases as well, which may have been responsible for the osteitis. Crizotinib can induce rapidly extensive osteitis, which can mimic tumor progression.

## Background

About 3–5% of non small cell lung cancer (NSCLC) harbor Anaplastic lymphoma kinase (*ALK)*-rearrangement, mainly with echinoderm microtubule-associated protein-like 4 (*EML4*) as a gene partner. The resulting fusion protein is constitutively activated and shows oncogenic properties [1]. Multiple ALK inhibitors are now available and were shown to improve patient outcomes [2–6]. The first ALK inihibitor to be approved was crizotinib, a small molecule tyrosine kinase inhibitor [7], which was the standard first-line treatment of patients with *ALK*-rearranged advanced NSCLC [8]. Ceritinib and alectinib are other ALK inhibitors available in several countries and alectinib is considered the new standard of care. Lorlatinib is also available in Japan [9] and brigatinib in the USA [10].

Crizotinib toxicity profile consists mainly in moderate visual disorders, electrocardiogram modification (mainly sinus bradycardia and QT interval prolongation), nausea, vomiting, diarrhea, liver enzyme elevation, fatigue, peripheral oedema, neutropenia and esophagitis [11, 12]. Here, we report the case of a previously undescribed side effect of crizotinib.

## Case presentation

**Fig. 1:**
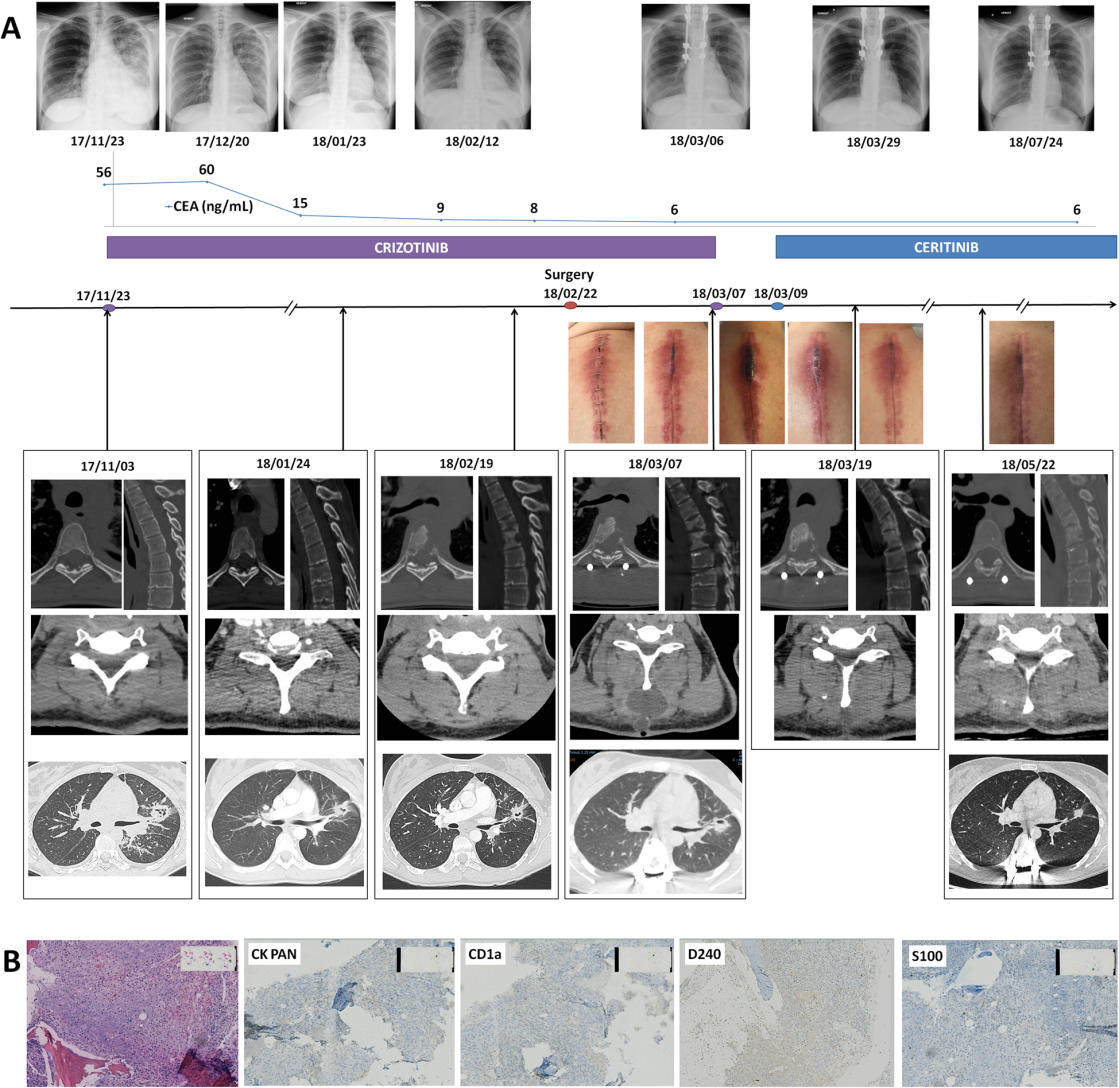
A necrotic bone lesion mimicking bone metastasis. **a** Chronology of treatments, CT-scan and MRI findings and photographs of the surgical suture. **b** Representative imaging of HES coloration and pan-cytokeratine, CD1a, D2–40 and PS100 immunostains of the bone biopsy sample

A 31-year-old woman with metastatic *ALK* rearranged NSCLC was referred to our center. There was no bone lesion at this time, neither on Computarized Tomography (CT)-scan nor on Fluoro-Desoxy-Glucose (FDG) Positron-EmissionTopmography (PET) scan. Crizotinib treatment was initiated as first line, with dramatic radiological response and rapid decrease of blood carcinoembryonic antigen (CEA) from 60 ng.mL-1 to 9 in 6 weeks (Fig. 1). Three months after crizotinib treatment onset, the patient presented with back pain, without neurologic disorder. She reported neither trauma nor pyrexia. She received no other medication during the previous 3 months. FDG-PET/CT scan revealed a hypermetabolic lesion on the 4th and 5th thoracic (Th4 and Th5) vertebrae. Spine Magnetic Resonnance Imaging (MRI) and CT-scan showed Th4 and Th5 vertebrae osteitis, anterior soft tissue infiltration and epiduritis. MRI T1 signal was highly enhanced after Gadolinium injection. Th4-Th5 spinal disc was intact. C Reactive Protein (CRP) level was 41 mg.L-1, white-blood cell count was normal, blood cultures were sterile, and CEA blood level remained at its lowest.

Because of the rapid extension of both osteitis and paravertebral collection that jeopardized spine stability, spinal cord decompression associated with histological removal and osteosynthesis were performed, by a posterior approach. Histological examination showed necrosis with abundant peripheral neutrophils, no microorganism nor malignant cell. Ziehl coloration was negative. Negativity of PS100 and CD1a immunostains eliminated Langherans cell histioctyosis. The absence of lymphatic vessel proliferation eliminated Gorham syndrome. Cultures of biopsy samples and Polymerase Chain Reaction (PCR) assays, including ribosomal 16S sequencing and mycobacterium complex PCR assay, remained negative. A week after surgery, severe inflammation and necrosis of the cutaneous surgical suture appeared, with subcutaneous and paravertebral soft tissue infiltration confirmed on CT-scan.

Because of the absence of tumour cell or germ on biopsy samples, crizotinib-induced osteitis was suspected. A retrospective review of the first chest CT-scan performed for assessment of tumor response to crizotinib 2 months after treatment initiation showed early signs of osteitis on the Th4 vertebra (Fig. 1). Crizotinib was suspended. The patient did not receive antibiotics. Subcutaneous inflammation regressed after 2 days. Ceritinib was initiated 2 days later. CT-scan at 2 weeks showed regression of osteitis and soft tissue infiltration. After 12 months, the patient is still on ceritinib, without any new lesion.

## Discussion and conclusions

Vertebral osteitis in a patient with stage IV cancer is commonly attributed to bone metastasis when infection has been ruled out. Nevertheless some differential diagnostic exist. Giving the fact that median overall survival in *ALK*-rearranged NSCLC reaches 4 years and even up to 81 months in the literature [13, 14], we believed that an invasive procedure was legitimate to rule out these differential diagnostic. In our patient’s case, diagnostic work-up ruled out an infectious osteitis, including tuberculosis. Several points made us confident that there was no bone metastasis: (i) the absence of malignant cell on surgical biopsies, (ii) the rapid regression of paravertebral collection after crizotinib withdrawal, before ceritinib treatment onset, (iii) the normal CEA blood level at the time of rapidly extensive bone and soft tissue lesion, while CEA blood level was elevated at the time of diagnosis and rapidly decreased thereafter. CEA use is not a recommended biomarker in NSCLC but was monitored in our patient. Other causes of osteitis have also been ruled out through histology and immunostainings analysis. Of note, unlike in Gorham’s vanishing bone syndrome or aneurysmal bone cysts, no marker of vascular proliferation or fibrosis was seen, suggesting a different entity. Lastly, non-specific inflammation cannot be ruled out, by definition. Nevertheless, the sequence of lesion improvement after crizotinib discontinuation support our hypothesis of a crizotinib-induced osteitis. Crizotinib is known to induce renal, pancreatic and liver cysts [8–12, 15, 16]. To our knowledge no crizotinib-induced osteitis has been reported to date.

Crizotinib differs from other ALK inhibitors as it potently inhibits other kinases, which may have been responsible for the osteitis. The favourable outcome despite ceritinib treatment is consistent with this hypothesis. Of note, crizotinib inhibits MET proto-oncogene (MET) and Macrophage Stimulating Factor 1 Receptor (MST1R, also peviously known as RON), two receptors that regulate osteogenesis through Platelet Derived Growth Factor (PDGF). PDGF pathway is thought to be involved in sorafenib- and imatinib- induced osteonecrosis by reducing the activity of osteoclastogenic cytokines including macrophage colonystimulating factor (M-CSF) and receptor activator of nuclear factor kappa-Β ligand (RANKL) [17]. PDGF signalling might have been involved in our patient’s osteitis. Other drug-induced osteonecrosis mechanisms are known such as Cyclooxygenoase 2 inhibition by non-steroid anti-inflammatory drugs and nuclear factor of activated T-cells cytoplasmic 1 (NFATc1) inhibition by bisphosphonates [18]. Crizotinib as no direct interaction with these pathways.

Crizotinib can induce rapidly extensive osteitis, which can mimic tumor progression. In our patient’s case, osteitis regressed after Crizotinib withdrawal and did not recur under ceritinib treatment.

## Data Availability

Not applicable.

## Abbreviations

ALK: Anaplastic lymphoma kinase
CEA: Carcinoembryonic antigen
CRP: C Reactive Protein
CT-scan: Computarized Tomography scan
EML4: Echinoderm microtubule-associated protein-like 4
FDG: Fluoro-Desoxy-Glucose
M-CSF: Macrophage colonystimulating factor
MET: MET proto-oncogene
MRI: Magnetic Resonnance Imaging
MST1R: Macrophage Stimulating Factor 1 Receptor (also peviously known as RON)
NFATc1: Nuclear factor of activated T-cells cytoplasmic 1
NSCLC: Non small cell lung cancer
PCR: Polymerase Chain Reaction
PDGF: Platelet Derived Growth Factor
PET-scan: Positron-EmissionTopmography scan
RANKL: Receptor activator of nuclear factor kappa-Β ligand
Th4 and Th5: 4th and 5th thoracic vertebrae
